# Molecular and morphological characterization of *Tylenchus zeae* n. sp. (Nematoda: Tylenchida) from Corn (*Zea mays*) in South Carolina

**DOI:** 10.2478/jofnem-2023-0003

**Published:** 2023-02-28

**Authors:** Mihail R. Kantor, Zafar A. Handoo, Sergei A. Subbotin, Joseph D. Mowery, Maria N. Hult, Stephen Rogers, Andrea M. Skantar

**Affiliations:** Mycology and Nematology Genetic Diversity and Biology Laboratory, USDA, ARS, Northeast Area, Beltsville, MD 20705 USA; Department of Plant Pathology and Environmental Microbiology, Pennsylvania State University, University Park, PA, 16802 USA; Plant Pest Diagnostic Center, California Department of Food and Agriculture, 3294 Meadowview Road, Sacramento, CA 95832 USA; Electron and Confocal Microscopy Unit, USDA, ARS, Northeast Area, Beltsville, MD 20705 USA

**Keywords:** 18S, 28S, *COI*, Corn, DNA sequencing, Molecular phylogeny, Morphology, Nematode, New species, Taxonomy, *Tylenchus zeae* n. sp.

## Abstract

Specimens of a tylenchid nematode were recovered in 2019 from soil samples collected from a corn field, located in Pickens County, South Carolina, USA. A moderate number of *Tylenchus* sp. adults (females and males) were recovered. Extracted nematodes were examined morphologically and molecularly for species identification, which indicated that the specimens of the tylenchid adults were a new species, described herein as *Tylenchus zeae* n. sp. Morphological examination and the morphometric details of the specimens were very close to the original descriptions of *Tylenchus sherianus* and *T. rex*. However, females of the new species can be differentiated from these species by body shape and length, shape of excretory duct, distance between anterior end and esophageal intestinal valve, and a few other characteristics given in the diagnosis. Males of the new species can be differentiated from the two closely related species by tail, spicules, and gubernaculum length. Cryo-scanning electron microscopy confirmed head bearing five or six annules; four to six cephalic sensilla represented by small pits at the rounded corners of the labial plate; a small, round oral plate; and a large, pit-like amphidial opening confined to the labial plate and extending three to four annules beyond it. Phylogenetic analysis of 18S rRNA gene sequences placed *Tylenchus zeae* n. sp. in a clade with *Tylenchus arcuatus* and several *Filenchus* spp., and the mitochondrial cytochrome oxidase c subunit 1 (*COI*) gene region separated the new species from *T. arcuatus* and other tylenchid species. In the 28S tree, *T. zeae* n. sp. showed a high level of sequence divergence and was positioned outside of the main *Tylenchus-Filenchus* clade.

According to the U.S. Department of Agriculture (USDA) National Agricultural Statistics Service (NASS), corn was the largest crop in the United States in 2019, with 91.7 million acres planted. Corn is a staple crop in South Carolina, and it was the most planted crop in 2019, with 380,000 acres (USDA NASS, 2019a, 2019b). The plant-parasitic nematodes that cause the most damage to corn grown in sandy soils include the spiral and root-lesion nematodes, followed by dagger, needle, ring, stunt, pin, lance, and stubby root nematodes (Tylka et al., 2011; Yan et al., 2016). Several other tylenchid nematode groups including representatives of the genus *Tylenchus* are reported as associated with corn. Most species of the genus *Tylenchus* are algal, fungal, and moss feeders (Yeates et al., 1993) or associated with grasses, scrubs, and tree roots (Yeates et al., 1993; Ciobanu et al., 2003). Geraert (2008) gave the history of the genus and updated genus diagnosis, list of valid species, identification key, and descriptions. Members of the genus *Tylenchus* Bastiian, 1865 are characterized by having a striated lip region, vulva situated far back after body center with the anterior ovary outstretched and post uterine branch short with elongate to filiform tails. The stylet is well developed with strong developed basal knobs (Thorne, 1962).

Molecular phylogenies including *Tylenchus* species have been based on 18S and 28S rDNA, and more recently on mitochondrial *COI* (Bai et al., 2020), but *T. arcuatus, T. naranensis*, and *T. davanei* are the only named species with representative sequences available (Bert et al., 2008; Ortiz, et al., 2016).

Nematodes of the genus *Tylenchus* sp. were recovered from soil samples collected from a corn field, located in Pickens County, South Carolina, USA, in 2019. The objective of this work was to provide morphological and molecular characterization of this nematode isolated from soil around corn in South Carolina, which is characterized herein as *Tylenchus zeae* n. sp.

## Materials and Methods

Two soil samples collected from a corn field from Pickens County, South Carolina, were sent by Diana Low (Clemson University) to the Mycology and Nematology Genetic Diversity and Biology Laboratory (MNGDBL) in Beltsville, Maryland, in fall of 2019 and early 2020. Nematodes were extracted from soil using the sugar centrifugal flotation method (Jenkins, 1964). For morphological study, nematodes were fixed in 3% formaldehyde and processed to glycerin by the formalin glycerin method (Hooper, 1970; Golden, 1990). Photomicrographs of the specimens were made with a Nikon Eclipse Ni compound microscope using a Nikon DS-Ri2 camera. Measurements were made with an ocular micrometer on a Leica WILD MPS48, Leitz DMRB compound microscope.

Cryo-scanning electron microscopy (Cryo-SEM) was used to obtain high-resolution images of females and males. Specimens previously mounted in glycerin on glass slides were carefully unmounted and rinsed in 50% ethanol. Individual nematodes were placed on a smooth Millipore membrane filter containing 0.4 μm pores, and excess ethanol was wicked through the membrane filter from below with filter paper. The membrane filter containing the nematodes was affixed to a copper plate, using conductive carbon tape and a small amount of cryo glue (Tissue Tek OCT Compound, Ted Pella, Inc., Redding, CA, USA), and conductively frozen for 30 sec on a precooled (–196°C) brass bar partially submerged in liquid nitrogen before the samples were fully submerged into liquid nitrogen. A vacuum was applied to the samples submerged in liquid nitrogen until the liquid nitrogen turned into a semisolid slush, and all air was evacuated from the cryo-transfer chamber. The samples were then transferred under vacuum into the cryo-prep chamber of the Quorum PP3010t Cryo Prep System (Quorum Technologies, Lewes, UK) and sublimated at –90°C for 15 min to remove any residual condensed water vapor from the surface of the samples. Following sublimation, the chamber temperature was lowered below –160°C, purged with argon, and the samples were coated with a 10 nm layer of platinum using a magnetron sputter head. After applying the conductive coating, the samples were transferred to a –175°C cryo-stage inside a Hitachi SU-7000 SEM (Hitachi High-Tech America, Inc., Dallas, TX, USA), and imaged using an accelerating voltage of 5 kV with a mixture of multiple electron detectors.

For molecular characterization, single nematodes were mechanically disrupted with a micro knife in 20 μl nematode extraction buffer (500 mM KCl, 100 mM Tris-Cl (pH 8.3), 15 mM MgCl_2_, 10 mM dithiothreitol (DTT), 4.5% Tween 20 and 0.1% gelatin; Thomas et al., 1997) and stored at –80°C until needed. To prepare DNA extract, frozen nematodes were thawed, 1 μl proteinase K (from 2 mg/ml stock solution) was added, and the tubes were incubated at 60°C for 60 min, followed by 95°C for 15 min to deactivate the proteinase K. Two or three microliters of extract were used for each PCR reaction. Five individuals were subjected to molecular analysis.

For characterization of *Tylenchus* sp., small subunit 18S rRNA (SSU) was amplified with primers SSU_F_04 (G18S4) (5’– GCTTGTCTCAAA GATTAAGCC – 3’) and SSU_R_81 (18P) (5’– TGATCCWKCYGCAGGTTC AC – 3’) (Blaxter et al., 1998) as described (Holtermann et al., 2006); additional sequencing primers included 550F (5’– GGCAAGTCTGGTGCCAG CAGCC – 3’), 1108R (5’– CCACTCCTGGTGGTGCCCTTCC – 3’). For individuals where the long fragment 18S was unsuccessful, primer 18S1.2 (5’– GGCGATCAGATACCGCCCTAG TT– 3’) and 18Sr2b (5’– TACAAAGGGCAGGGACGTAAT– 3’) were used. The D2-D3 expansion segments of 28S rRNA gene were amplified with primers D2A (5’–ACAAGTACCGTGAGGGAAAGTT – 3’) and D3B (5’– TCGGAAGGAACCAGCTACTA – 3’) (De Ley et al., 2005) as described previously (Skantar et al., 2021). The mitochondrial *COI* gene was amplified with primers JB3 (5’– TTTTTTGGGCATCCTGAGGTTTAT–3’) and JB5 (5’– AGCACCTAAACTTAA AACATAATGAAAATG– 3’) according to Derycke et al. (2010). All PCR products were cleaned with the Monarch DNA Gel Extraction Kit (NEB, Ipswich, MA) and sequenced directly by Genewiz, Inc. The sequences were submitted to GenBank as follows: 18S, MZ330373; 28S, MZ330374; *COI*, MZ332970–MZ332971.

DNA sequences of 18S and 28S D2-D3 rRNA and mitochondrial *COI* genes from the *Tylenchus* sp. were analyzed by BlastN to identify similarity to those in GenBank. Alignments were made with DNA sequences from selected species using MAFFT or Clustal Omega modules within Geneious Prime (2020.1.0). Phylogenetic analysis was conducted by Bayesian inference (Huelsenbeck and Ronquist, 2001) via the CIPRES Gateway (Miller et al., 2010) plug-in in Geneious. The model of nucleotide evolution for 18S, 28S, and *COI* was determined with jModelTest 2.1.7 (Darriba et al., 2012) to be GTR + I + G, according to Akaike’s information criteria. Bayesian inference was run with random starting trees, four chains for 2 × 10^6^ generations, with Markov chains sampled every 200 generations. Two runs were performed for each analysis. Burn-in samples were discarded, and convergence was evaluated, with remaining samples retained for further analysis. Topologies were used to generate 50% majority rule consensus trees with posterior probabilities shown on appropriate clades.

## Systematics

**Figure 1:**
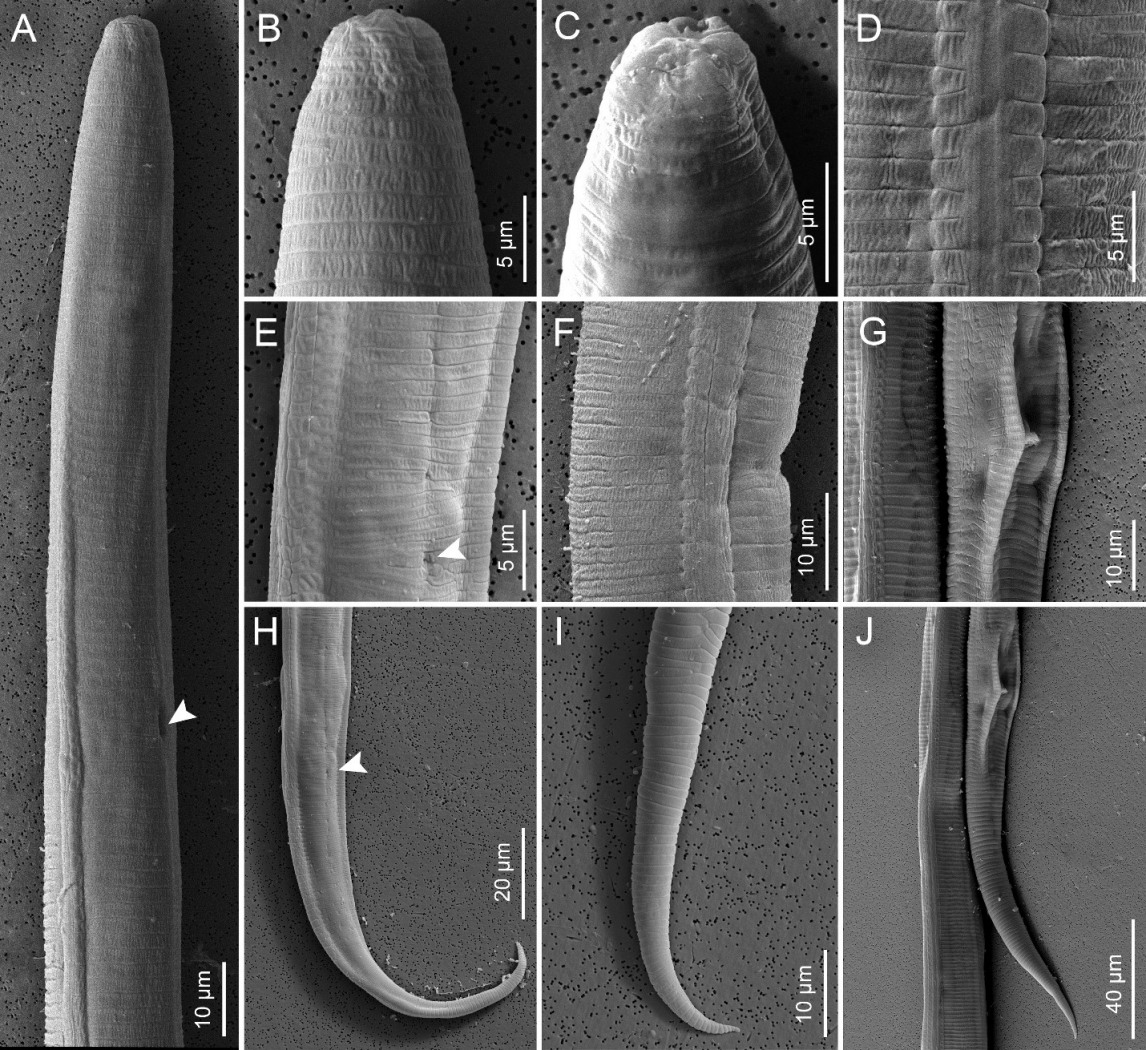
Scanning electron micrograph (SEM) images of *Tylenchus zeae* n. sp. A: Female specimen, anterior end, arrow pointing toward the excretory pore; B: Female specimen, head; C: Female specimen, face view; D: Lateral field (midbody); E: Female specimen, anal opening; F: Female specimen, vulval opening; G: Male specimen, spicule; H: Female specimen, arrow showing the anal opening; I: Female specimen, tail; J: Male specimen, posterior end.

**Figure 2:**
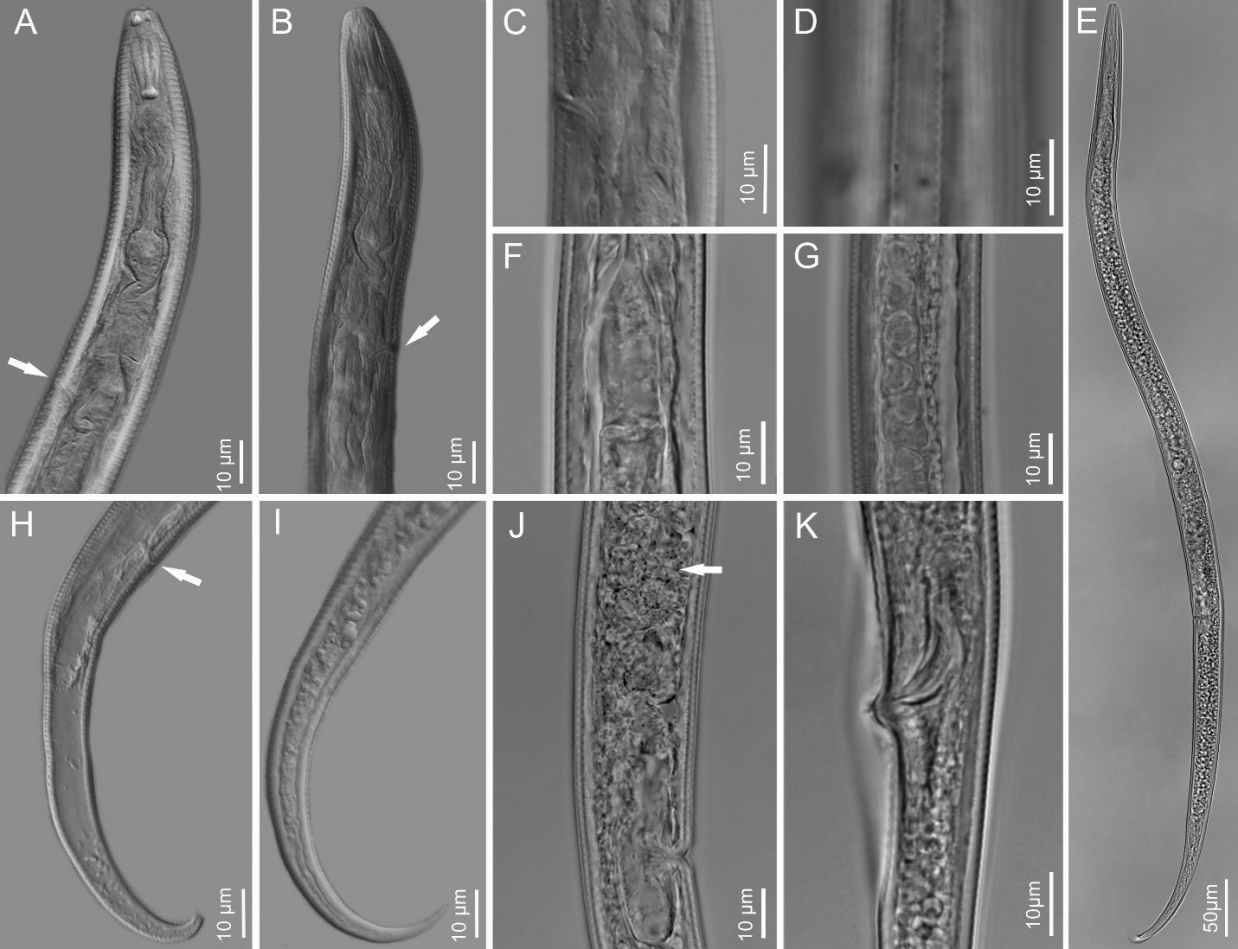
Photomicrographs of *Tylenchus zeae* n. sp. males and females. A–B: Anterior end with arrows pointing toward the excretory pore; C: Excretory pore; D: Areolated lateral field; E: Entire female body; F: Female basal bulb; G: Female gonad; H–I: female posterior end with arrow pointing the anal area (H); J: Female vulva region with arrow pointing toward the spermatheca; K: Male spicule.

**Figure 3:**
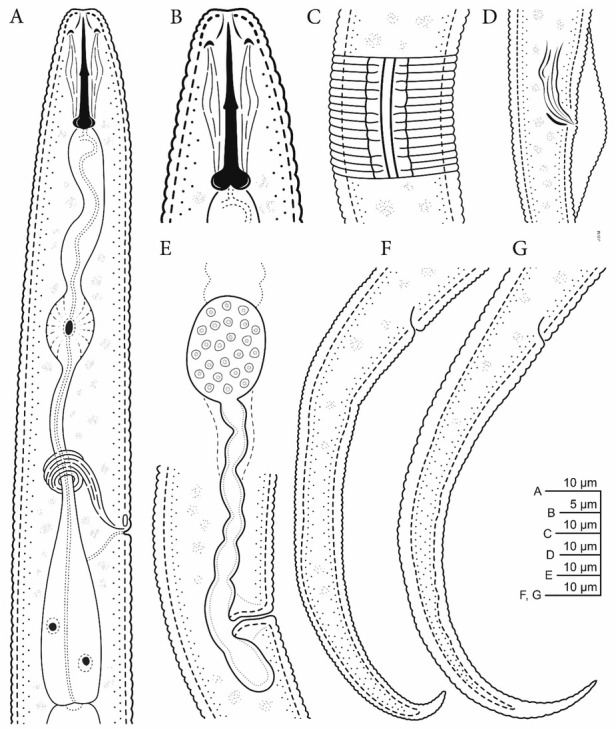
Line drawings of *Tylenchus zeae* n. sp. A: Female pharyngeal region; B: Female lip region showing stylet; C: Areolated lateral field; D: Male spicule, gubernaculum, and bursa. E: Vulval region showing vulva, uterus, and spermatheca; F–G: female tails.

**Figure 4:**
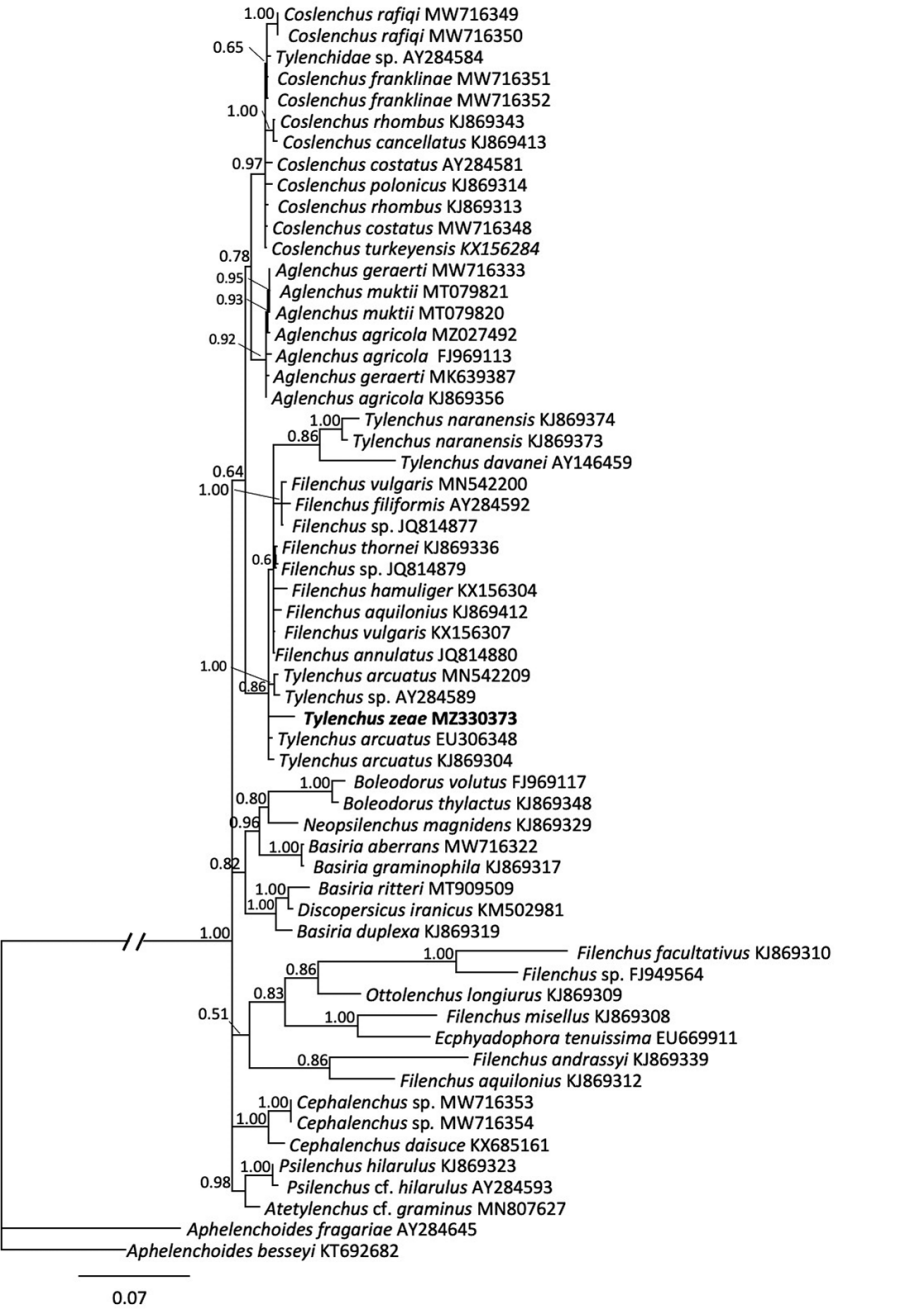
Phylogenetic relationships of *Tylenchus zeae* n. sp. with other select Tylenchidae, as inferred from a 1585 bp alignment of 18S rRNA sequences, according to the GTR + I + G model of nucleotide substitution and incorporated into MrBayes (MB) as described. A 50% majority rule consensus tree was generated with posterior probabilities (PP) shown on appropriate branches, with *Aphelenchoides besseyi* as the outgroup. New sequences are indicated in bold.

**Figure 5:**
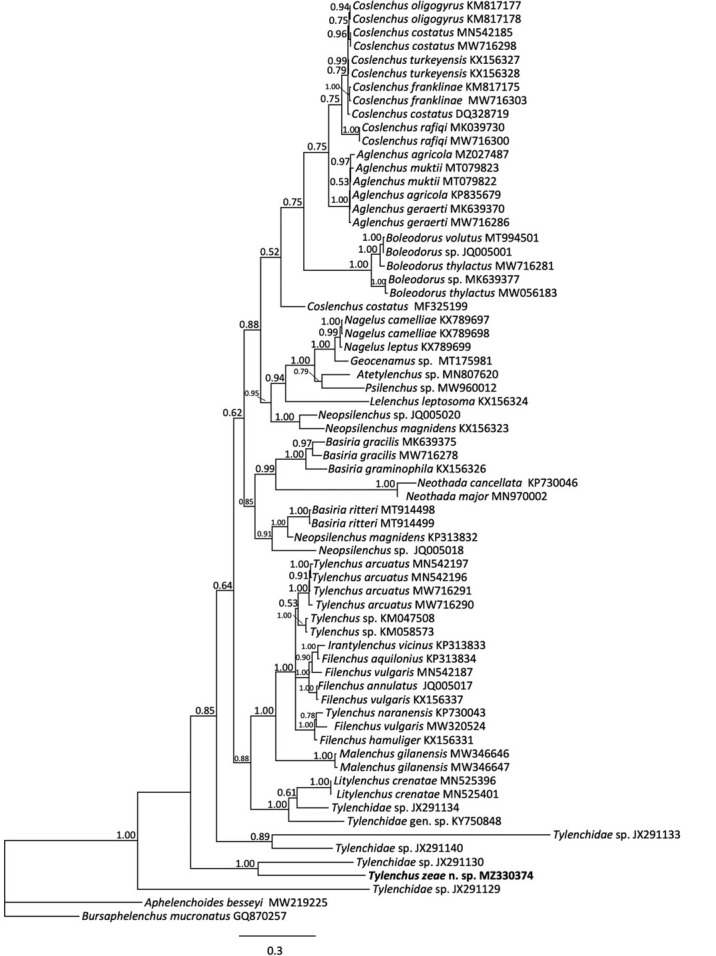
Phylogenetic relationships of *Tylenchus zeae* n. sp. with other select Tylenchidae, as inferred from an 822 bp alignment of 28S rRNA sequences, according to the GTR + I + G model of nucleotide substitution and incorporated into MrBayes (MB) as described. A 50% majority rule consensus tree was generated with posterior probabilities (PP) shown on appropriate branches, with *Bursaphelenchus mucronatus* as the outgroup. New sequences are indicated in bold.

*Tylenchus zeae* n. sp. (Figs. 1-5).

## Measurements

Measurements are given in Table 1.

**Table 1:** Morphometrics of *Tylenchus zeae* n. sp. All measurements are in μm and in the form: mean ± standard deviation (range).

Character	Holotype	Female (*n* = 9)	(Male *n* = 4)
L	830	830.0 ± 39.3 (765.0–895.0)	813.0 ± 45 (775.0–885.0)
Stylet	20	20.0 ± 0.7 (20.0–22.0)	20.0 (20.0–20.0)
MBW	27	25.0±2.58 (20.0–30.0)	22.0 (20.0–25.0)
Ant. end to exc. pore distance	102	103.0 ± 6.0 (92.0–115.0)	—
Ant. end to esophago-intestinal valve	122	123.0 ± 5.5 (115.0–130.0)	123.0 ± 2.0 (120.0–125.0)
Tail	112	121.0 ± 5.9 (112.0–127.0)	127.0 ± 5.0 (122.0–135.0)
a	31	33.0 ± 3.7 (28.0–42.0)	37.0 ± 2.0 (35.0–39.0)
b	7	7.0 ± 0.5 (6.0–8.0)	7.0 ± 0.4 (6.0–7.0)
c	7	7.0	6.0 ± 0.3 (6.0–7.0)
V%	63	63.0 ± 1.2 (61–65%)	—
Anal body width	15	15 ± 1.28 (12.0–17.0)	13.0 (12.0–14.0)
Spicules	—	—	21.0 ± 1.0 (20.0–23.0)
Gubernaculum	—	—	6.0 ± 0.5 (5.0–6.0)

L, body length; MBW, maximum body width.

## Description

*Female*: The female body is vermiform, assuming arcuate C-shaped form when killed by gentle heat. Cuticle is strongly striated. The lip region is striated, and the head almost continues but is distinctly offset and bearing five or six annules. In SEM (Fig. 1) four to six cephalic sensilla were noticed, represented by small pits at the rounded corners of the labial plate. The oral plate is small and round. The amphideal opening is large and pit-like, confined to the labial plate, extending three or four annules beyond the labial plate. The stylet is heavy and well developed, between 20 and 22 μm long. The conus is half of the stylet length. Basal knobs are rounded, well developed, and sloping posteriorly. The median bulb is large and oval, with an elongated basal bulb. The Excretory pore is visible and the excretory duct is heavily sclerotized. Four wide lines in the lateral fields are characteristic with crenate outer margins (Figs. 2D; 3C) and outer bands areolated with areolation mostly not joining. The female gonad is long and outstretched, almost reaching to the pharynx area with round to oval spermatheca filled with large round sperms. Vulva lips were not protruding but occasionally noticed in a few specimens slightly protruding. The vagina is about one third of the body width at vulva. The post vulval uterine sac is more than half of the vulval diameter. The tail is slender, almost cylindrical, regularly tapering, and arcuate with its terminus finely to bluntly rounded. Tail annulation is prominent, extending to the tail terminus.

*Male*: The male is similar to females in general body characteristics, except for the reproductive organs. Spicules are long, curved ventrally, and gubernaculum is small. Bursa adanal is short. Tail is an elongate conoid, with a tail terminus bent ventrally, and finely rounded.

## Diagnosis and relationships

*Tylenchus zeae* n. sp. females are similar to *Tylenchus rex* Andrassy, 1979 from which they differ by having a slightly different body shape (vermiform, assuming arcuate C shaped vs. body almost straight, hardly curved), shorter body length of 830 (765–895) μm vs. 1,007 (960–1009) μm, a basal bulb that is long and moderately developed, elongated vs. fairly small, spoon-shaped, and a shorter distance from anterior end to excretory pore (103 vs. 130 μm), as well as the shorter tail length (112–127 μm vs. 127–160 μm) with tail not showing two slightly visible breaks and tail terminus not pointed vs. rounded when compared to the *T. rex* population reported by Andrassy (1979). The a, b, c, and V% (Table 2) values are within the range reported by Brzeski (1996). Males of *T. zeae* n. sp. have longer spicules, 23 to 27 μm vs. 20 to 23 μm.

**Table 2:** Measurements for *Tylenchus zeae* n. sp. comparison with *T. rex* and *T. sherianus* female populations. Measurements are in μm and in the form: mean ± standard deviation (range).

Character	*T. zeae* n. sp. (*n* = 9)	*T. sherianus* Andrassy (1979)	*T. rex* Brzeski (1996) (*n* = 12)	*T. rex* Andrassy (1979)	*T. rex Geraert* (2008)
L	830.0 ± 39.3 (765.0–895.0)	750–840	1007.0 ± 52.4 (963.0–1087.0)	960.0–980.0	960–1009
Stylet	20.3 ± 0.7 (20.0–22.0)	19–20	20.4 ± 0.4 (19.5–205)	20.0–21.0	19–21
MBW	26.0 (20.0–30.0)	—	30.7 (based on author calculations)	—	—
Ant. to exc. pore distance	103.0 ± 6.0 (92.0–115.0)	—	130.0 ± 4.9 (121.0–157.0)	—	—
Ant. to esophago-intestinal valve	123.0 ± 5.5 (115.0–130.0)	—	151.0 ± 4.4 (142.0–157.0)	—	133–157
Tail	120.0 ± 5.9 (112.0–127.0)	100–116	134.0 ± 4.7 (127.0–141.0)	134.0–160.0	127–160
a	33.0 ± 3.7 (28.0–42.0)	25–28	32.8 ± 1.9 (30.0–36.0)	32.0–34.0	—
b	7.0 ± 0.5 (6.0–8.0)	5.7–6.0	6.7 ± 0.3 (6.4–7.3)	6.8–7.0	—
c	7.0	6.8–8.0	7.5 ± 0.4 (7.0–8.4)	6.0–7.1	6.0–8.4
V%	63.0 ± 1.2 (61–65%)	65–68	64.9 ± 0.8 (63.0–66.0)	61.0–63.0	61–66
MB%	—	43	44.2 ± 0.8 (43–45)	43–45	43–45

L, body length; MBW, maximum body width.

*Tylenchus zeae* n. sp. females are close to *T. sherianus* Andrassy, 1981, although they differ from the latter by the shape of the median bulb, which is oval in *T. zeae* n. sp. compared with a more rhombus shape in *T. sherianus*; longer stylet length (20–22 μm vs. 19–20 μm); a higher a value (28–42 vs. 25–28); and a b value that is also slightly larger (6–8 vs. 5–7); and a slightly lower V% (61–65% vs. 65–68%). The tail length is within the range (112–127 vs 100–116 μm). The shape of the excretory duct is different in *T. zeae* n. sp. when compared to *T. sherianus*, in which the duct is doubled curved, forming an S shape, whereas in *T. zeae* n. sp. it is heavily sclerotized and straight. The lateral field has four lines that are characteristic with crenate outer margins and outer bands areolated with areolation mostly not joining vs. a lateral field with four narrow, smooth lines with no crenated margins or areolations in *T. sherianus. T. zeae* n. sp. have a bluntly rounded tail terminus, vs. a pointed terminus in *T. sherianus*. Males of *T. zeae* n. sp. have a shorter tail (122–135 μm vs. 155 μm) than given for *T. sherianus* in the original description and it is sharply pointed. The gubernaculum is also slightly shorter in *T. zeae* n. sp. (5–6 μm vs. 8 μm).

The other two species that *T. zeae* n. sp. comes close to are *Tylenchus davainei* Bastian, 1865 and *Tylenchus arcuatus* Siddiqi, 1963. *Tylenchus zeae* n. sp. differs from *Tylenchus arcuatus* by having a longer stylet (20–22 μm vs. 15–17.5 μm). The a value is much higher in *T. zeae* n. sp. (28–42 vs. 23–29 in *T. arcuatus*), the b value for *T. zeae* n. sp. is 7.0 (6–8), whereas for *T. arcuatus* it is 6.0 (4.9–6.0). *T. zeae* n. sp. have a smaller V% (61–66% vs. 64–71%) and a longer tail (112– 160 μm vs. 80–137 μm) that is finely rounded vs. robust, with a hooked, sharply broken, pointed tip. Females differ from *T. davainei* by having a longer stylet (20.0– 22.0 μm vs. 15.8–17.6 μm) with rounded stylet knobs vs. anteriorly directed knobs, and a slightly shorter body length (765.0–895.0 μm vs. 863.0-1182.0 μm). Males of *T. zeae* n. sp. have a larger a value (35.0–39.0 vs. 28.9-35.2) than *T. davainei*.

## Type host and locality

*T. zeae* n. sp. is associated with roots and soil of corn (*Zea mays*) in Pickens County, South Carolina. The Global Positioning System coordinates are 35.00341 N, 82.65306 W.

## Type material

*Holotype (Female)*: Slide T-766t, deposited in the United States Department of Agriculture Nematode Collection, Beltsville, MD, USA.

*Paratypes (Females and Males)*: Same data and repository as holotype. Slides T-7629p–T-7635p.

*Zoobank ID*: urn:lsid:zoobank.org:act:430F0F46-8163-4636-8FC3-97D90A0B0519

## Etymology

The species name is derived from *Zea mays*, the host of this species.

## Molecular phylogenetic analysis

Amplification and sequencing of 18S rRNA resulted in a 1605 bp fragment from PCR with primers SSU_F_04 and SSU_R_81 from one individual. For three other nematodes, primers 18S1.2 and 18Sr2b were used to generate fragments of 555 to 575 bp. Because the longer fragment contained more variable sites for comparison, that sequence was used for subsequent phylogenetic analysis. BlastN comparison to sequences in GenBank resulted in the highest similarity to *Filenchus vulgaris* (KX156307), differing at 32 bp (2.24%) and to *Tylenchus arcucatus* (EU306348), differing at 36 bp (2.24%). For the 18S rRNA gene, 59 sequences from 44 taxa were used to construct a 1598 bp alignment used for Bayesian inference. In the 18S tree (Fig. 4), *T. zeae* n. sp. and two sequences from *T. arctuatus* grouped together but were unresolved. All three of these sequences appeared within a larger clade that included a third *T. arcuatus* sequence, an unspecified *Tylenchus* sp., several *Filenchus* spp., and a subclade containing *T. naranensis* and *T. davanei*.

For 28S rRNA, amplification from five individuals resulted in a 738 bp fragment. Sequences obtained from PCR of four of five specimens were of insufficient quality to generate independent contigs, although partial reads did match the full-length D2-D3 obtained from the high-quality sequence. The representative sequence used for subsequent analysis was 79.77% similar to one from an undescribed *Tylenchus* sp. CD207 from palm soil in Florida (JX291130), differing at 140 bp. The highest pairwise similarity among all sequences available in GenBank (82.95%) was to *Helicotylenchus oleae* (MF287653). For 28S phylogenetic analysis, 69 Tylenchidae sequences were used to construct an 822 bp alignment. In the inferred 28S MrBayes tree (Fig. 5), the *Tylenchus zeae* n. sp. grouped with an undescribed Tylenchidae sp. (JX291130) with strong support (PP = 1.00). However, the genus did not form a monophyletic group, with *T. arcuatus* and other *Tylenchus* populations forming clades with *Filenchus* species and others with *Litylenchus crenatae*. This finding is consistent with the polyphyletic groupings observed by others (Atighi et al., 2013; Qing et al., 2017; Qing and Bert, 2019; Bai et al., 2020).

**Figure 6:**
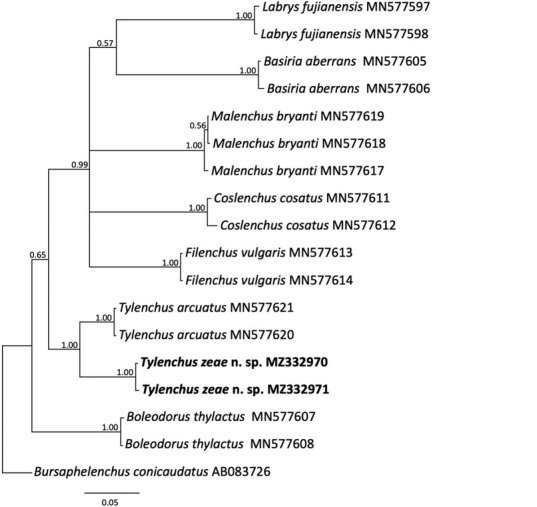
Phylogenetic relationships of *Tylenchus zeae* n. sp. with other select Tylenchidae, as inferred from a 418 bp alignment of mitochondrial *COI* sequences, according to the GTR + I + G model of nucleotide substitution and incorporated into MrBayes (MB) as described. A 50% majority rule consensus tree was generated with posterior probabilities (PP) shown on appropriate branches, with *Bursaphelenchus conicaudatus as* the outgroup. New sequences are indicated in bold.

For mitochondrial *COI*, fragments of 416 bp were amplified from two nematodes, differing from each other at a single nucleotide. Sequence similarity was highest (90.38%) with *Tylenchus arcuatus* from China (MN577620), differing at 40 bp (9.6%). The *COI* gene tree contained 18 sequences from fewer taxa but with strong branch support for individual clades; the SC population appeared as a sister group to *T. arcuatus* and was clearly resolved from *Filenchus vulgaris* (Fig. 6). A relative lack of available *COI* gene sequences prevented further cross-comparison of the *T. zeae* n. sp. position relative to most other species present within the 18S rRNA gene tree. *Tylenchus zeae* differs from its nearest neighbor in the 18S tree, *T. arcuatus*, at 2.5% pairwise difference and at 9.9% in the *COI*. In comparison, *F. vulgaris*, which is also poorly resolved from *T. arcuatus* in the18S tree, differs at 0.6% in this gene and at 16.1% in *COI*. Although not perfect parallel comparisons, they do give some measure of the relative sequence diversity in 18S and *COI*, as reflected in the separate species.

## Discussion

In this study, a new species of the family Tylenchidae is herein described and illustrated based on morphological, morphometric, and molecular characters. Molecular phylogenies of the Tylenchidae have primarily included SSU 18S and LSU 28S rDNA (Atighi et al., 2013; Qing et al., 2017; Qing and Bert, 2019), although some recent studies have included the mitochondrial *COI* barcode (Bai et al., 2020; Mortazavi et al., 2021).

To date, only a few *Tylenchus* species have been characterized molecularly, including *T. naranensis, T. davanei*, and *T. arcuatus*, which is not surprising considering the challenging taxonomy of the group. *Tylenchus zeae* n. sp. is unresolved from *T. arcuatus* in the 18S tree (Fig. 4). *Tylenchus naranensis* and *T. davanei* grouped together in a moderately supported (PP = 0.86) clade separate from *T. zeae* n. sp. In contrast, within some other 18S or multigene trees, *T. davanei* clusters with *F. andrassyi* and *F. aquilonius* (Qing et al., 2017; Munawar et al., 2021). This difference in placement could be due to shorter 18S alignments used in those studies. *Filenchus* is polyphyletic in our 18S tree, in agreement with Qing et al. (2017), and *Tylenchus zeae* n. sp. groups with species of *Filenchus* within Clade 1 (as defined by Qing et al., 2017), which includes taxa characterized by four incisures in the lateral field (Qing and Bert, 2019). Morphologically, *T. zeae* n. sp. fits within this placement (Figs. 1D; 2C). SEM imaging confirmed head bearing five or six annules, four to six cephalic sensilla represented by small pits at the rounded corners of the labial plate; a small, round oral plate; a large, pit-like amphidial opening confined to the labial plate and extending three or four annules beyond the labial plate.

Phylogenetic analysis of the 28S rRNA gene sequence alignment grouped *T. zeae* n. sp. with several undescribed *Tylenchidae* spp. apart from *T. arcuatus* (Fig. 5), in contrast with their close relationships shown by the other two markers examined. Qing and Bert (2019) also noted a discordance between 18S and 28S rRNA gene trees for the Tylenchidae, indicating the possibility variable copies or pseudogenes were amplified from nematodes due to PCR and primer bias. As shown in previous studies (Bai et al., 2020), the genera within Tylenchidae were not always monophyletic in 28S rRNA gene, and for the *COI* gene trees in many cases, clades were weakly supported. Limitations of ribosomal markers were also noted in the phylogenetic analysis of *Malenchus* and *Filenchus* by Qing et al. (2017) and in the broader Tylenchidae phylogenies of Qing and Bert (2019). A relatively high substitution rate in 28S rRNA can also lead to long-branch attraction that could obscure phylogenetic relationships. Within early diverging groups, resolution is low (Bert et al., 2008; van Megen et al., 2009), which is a problem not resolvable by simply adding taxa (Qing et al., 2019). Moreover, the amount of rRNA polymorphism that exists in Tylenchidae has not been sufficiently explored. It also needs to be noted that for many genera, there are no sequences represented in GenBank, particularly for *COI*. Many species are only represented by a single sequence, which limits the comparisons of performance of the different genes.

Some of the species with similar characteristics to *T. zeae* n. sp. are *Tylenchus davainei* Bastian, 1865, which is a cosmopolitan species, found not only in soil but also in aquatic environments (Geraert, 2008). *Tylenchus arcuatus* Siddiqi, 1963 is described from Simla, India, and reported from France, Hungary, California (Andrassy, 1979), Poland, Switzerland, Iowa (Brzeski, 1996), the Netherlands (Bongers, 1988), and Belgium (Bert and Geraert, 2000). *Tylenchus rex* Andrassy, 1979 has a limited distribution and has been reported only three times from two different continents (Brzeski, 1996; Geraert, 2008). Originally it was described by Andrassy (1979) from moss collected on the slopes of Ben Hedi, Scotland. The other reports of this species came from Poland and Australia by Brzeski (1996). *Tylenchus sherianus* Andrassy, 1981 is reported only from Cameroon. In 1981, while revising the genus *Tylenchus* (pp. 27, 29), Andrassy described among others a new species *Tylenchus sheri* Andrassy, 1979 and then coincidentally Andrassy received a publication from Khan and Khan (1978) in which they also described a new species under the same name *Tylenchus sheri*, Khan and Khan 1978. Because the earlier publication date takes priority for the name, Andrassy (1981) then proposed in 1981 a new name for his species (*Tylenchus sherianus* nom. new = *Tylenchus sheri* Andrassy, 1979, nec Khan & Khan, 1978).

Although molecularly *Tylenchus* and *Filenchus* genus group together, including morphologically some species of *Tylenchus* are synonymized with *Filenchus*. However, in *Filenchus* the stylet is moderately developed, and the length is generally short (7–15 μm long); conus is solid and appears anteriorly, sharply pointed, about one third or less of the total stylet length; in *Tylenchus*, the stylet is longer (8–21 μm long), with conus about half of the stylet length. In addition, the larger pit-like amphidial opening is generally confined to the labial plate in *Tylenchus* vs. elongated, slit-like apertures extending three or four annuli beyond the labial plate in most of the *Filenchus* species. Accordingly, the current species (*T. zeae* n. sp.) with most of these differences fits better in the genus *Tylenchus*.

Based on the collective morphological and molecular analysis, this population from South Carolina is characterized as *Tylenchus zeae* n. sp. It should be noted that the overall picture within the family Tylenchidae remains complicated. Further morphological and molecular investigations of underrepresented species are needed to strengthen identifications and phylogenetic relationships among these nematodes.
